# Early experience on omaveloxolone in adult patients with Friedreich’s ataxia: a real-world observational study

**DOI:** 10.1007/s00415-025-13487-1

**Published:** 2025-11-01

**Authors:** Salvatore Maria Lima, Marta Caltagirone, Christian Messina, Umberto Quartetti, Nicasio Rini, Flora D’Amico, Filippo Brighina, Vincenzo Di Stefano

**Affiliations:** 1 Regional Center for Diagnosis and Treatment of Neuromuscular Disease, Department of Biomedicine, Neuroscience, and Advanced Diagnostic (BIND), University of Palermo, Via del Vespro 143, 90129 Palermo, Italy; 2 Azienda Sanitaria Provinciale Catania, Catania, Italy

**Keywords:** Friedreich’s ataxia, Spinocerebellar ataxia, FRDA, Frataxin, Omaveloxolone, MFARS, Nrf2, Mitochondria

## Abstract

**Introduction:**

Friedreich’s ataxia (FRDA) is an autosomal recessive neurodegenerative spinocerebellar ataxia caused by a homozygous GAA triplet repeat expansion in the frataxin (FXN) gene. FRDA is a multisystem disorder involving the central and peripheral nervous systems, the musculoskeletal system, the heart, and the endocrine pancreas. In recent years, Omaveloxolone, a potent activator of nuclear factor erythroid 2-related factor 2 signaling, showed a significant neurological improvement compared to placebo, with a good safety profile. With this study, we report an early real-life experience on a cohort of FRDA patients treated with omaveloxolone.

**Materials and methods:**

Patients were assessed with an anamnestic profile, general and neurological examination, clinical scales (mFARS, SARA, and FA-ADL) and blood tests, at baseline, at 12 weeks and after 24 weeks of treatment. Inclusion criteria were genetical diagnosis of FRDA, age ≥ 18 years and mFARS < 80. Exclusion criteria included severe hepatic and renal impairment, and severe heart failure. Each patient received oral omaveloxolone at a dose of 150 mg/day.

**Results:**

Twenty patients (65% females) affected by FRDA aged 40.6 ± 12.6 years and a duration of disease of 24.9 ± 9.5 years were treated with omaveloxolone and followed up for 25.2 ± 8.0 weeks. The drug was safe with no significant adverse events during the first 24 weeks and without discontinuations. Indeed, asymptomatic, and transient liver transaminase elevation occurred in 50% of patients. Cardiac function was stable, as well as NT-proBNP and lipids. Clinical scales did not show any significant difference during follow-up, but a significant reduction in IL-6 was reported.

**Conclusions and discussion:**

Omaveloxolone seems to be safe and well-tolerated in adult FRDA patients in the real-life setting. No significant worsening of symptoms was observed with no signs of progression, as well as the improvement of inflammatory biomarkers after 24 weeks of treatment, but no predictive factors for the disease response have been identified. However, the short duration, and the small sample size limit the generalizability of the results. Further studies with longer observation are needed to clearly define the efficacy of omaveloxolone in FRDA.

**Supplementary Information:**

The online version contains supplementary material available at 10.1007/s00415-025-13487-1.

## Introduction

Friedreich’s ataxia (FRDA) is an autosomal recessive neurodegenerative spinocerebellar ataxia, and the most common inherited ataxia in Europe [1, 2]. In most cases, the disease is caused by a homozygous GAA triplet repeat expansion in the frataxin (FXN) gene, with repeat lengths correlating with onset and disease severity [1]. The diagnosis is established by the finding of expanded GAA repeats (GAA > 66) in both copies of the FXN gene [2]. FRDA is a multisystem disorder that typically manifests between the ages of 10 and 16 years, involving the central and peripheral nervous systems, the musculoskeletal system, the heart, and the endocrine pancreas [1]. The ‘classical’ FRDA phenotype is usually characterized by gait and limb ataxia, dysarthria, loss of lower limb reflexes with deep sensory loss, Babinski sign and pes cavus, as well as non-neurological signs, including hypertrophic cardiomyopathy and diabetes mellitus [1, 2]. However, a significant number of cases are classified as 'atypical', with most falling into the category of delayed-onset forms, namely, late-onset Friedreich’s ataxia after age 25, and very late-onset FRDA after age 40 [1]. Disease progression is commonly assessed using clinical scales such as the modified Friedreich’s Ataxia Rating Scale (mFARS), the Scale for the Assessment and Rating of Ataxia (SARA), which largely overlap but differ in certain key aspects, such as the inclusion of functional measures [1]. Notwithstanding the recent advances in the study of FRDA patients, the mechanisms by which FXN deficiency leads to impaired bioenergetic efficiency in the cells of FRDA patients remains unclear. FXN has been proposed to participate in different capacities, such as iron chaperone during cellular heme and iron–sulfur (Fe–S) cluster production and iron-storage protein during conditions of iron overload. Furthermore, FXN plays a crucial role in the mitochondrial biogenesis of iron–sulfur (Fe–S) clusters, which serve as essential redox cofactors for multiple enzymes, including those of the respiratory chain complexes I, II, and III. Through its involvement in Fe–S cluster assembly, FXN contributes to a wide range of cellular processes, such as mitochondrial energy production, redox catalysis, β-oxidation of fatty acids, regulation of gene expression, and maintenance of cellular iron homeostasis via direct iron binding. Moreover, Fe–S-dependent enzymes are key players in DNA repair and replication, linking frataxin deficiency to genomic instability and cellular dysfunction [3, 4]. In the past years, several molecules have been investigated for their potential to counteract the downstream effects of FXN deficiency, with antioxidant and iron chelation agents being evaluated both as monotherapy and in combination, yielding inconsistent results [1]. Although no approved treatment currently exists for FRDA, the use of antioxidants such as vitamin E and coenzyme Q10 remains widespread among patients [5]. Despite their proposed benefits on cardiac and skeletal energy metabolism, these supplements have not demonstrated efficacy in blinded clinical trials [5]. Although some supplements or antioxidants, such as idebenone, showed modest benefits in open-label studies, they have failed to meet registration-level endpoints in controlled clinical trials [5]. In addition, FXN deficiency disrupts antioxidant defenses, potentially contributing to disease progression through a vicious cycle involving mitochondrial dysfunction, impaired nuclear factor erythroid 2-related factor 2 (Nrf2) signaling, and reduced ATP production [6]. In healthy conditions, oxidative stress triggers Nrf2 translocation to the nucleus, promoting the expression of antioxidant genes and cellular protection [6]. In FRDA, both mitochondrial activity and Nrf2 signaling are compromised [6]. Omaveloxolone is a potent activator of Nrf2 [6]. In vitro, omaveloxolone has been shown to restore mitochondrial function in fibroblasts from FRDA patients and in neurons from various FRDA mouse models [6]. Its safety and efficacy were assessed in the multicenter, double-blind, placebo-controlled MOXIe trial (NCT02255435), where treatment led to significant neurological improvement compared to placebo and was generally well-tolerated [6]. Abnormalities in serum liver function tests were confined to transaminases, resolved upon dose interruption or reduction, and significantly decreased over time [7]. Therefore, the safety profile of omaveloxolone in clinical practice is expected to mirror the favorable outcomes observed in clinical trials [7]. However, as omaveloxolone was approved for Friedreich ataxia since July 2023 [7], there is still a lack of real-life studies, and the limited evidence comes from clinical trials with very low sample size and case reports [8, 9]. The present study describes the early experience on real-life setting on the use of omaveloxolone in a cohort of adult FRDA patients, reporting clinical and laboratory outcomes after 6 months of treatment.

## Materials and methods

### Ethics and approval

The study was approved by the competent Ethical Committee (“Comitato Etico Locale Palermo 1”) on 10th December 2024 (V n.28/2024), and it was conducted in conformity with the Declaration of Helsinki principles. Written consent was obtained from each patient for the use of anonymized clinical data for research purposes.

### Study design and inclusion criteria

We conducted a single-center, observational, prospective study to evaluate the safety and efficacy of omaveloxolone in patients with FRDA in a real-world clinical setting. Starting on the 2nd January 2025 we enrolled patients with a clinical and genetic diagnosis of FRDA who were followed at the Regional Center for Neuromuscular Rare diseases at University Hospital P. Giaccone of Palermo. Patients were considered eligible for inclusion if they met the following criteria:

- Diagnosis of FRDA, confirmed through genetic testing.
- Treatment with omaveloxolone as part of clinical practice.
- mFARS score < 80 at study initiation.
- Age ≥ 18 years at the time of informed consent and treatment initiation.
- A minimum follow-up of 4 weeks from omaveloxolone start.

Exclusion criteria included:

- mFARS score ≥ 80 at study initiation
- severe hepatic impairment (Child–Pugh class C)
- estimated glomerular filtration rate (eGFR) < 30 mL/min
- heart failure with New York Heart Association (NYHA) functional class > III.

Each patient received oral omaveloxolone at a dose of 150 mg/day, administered as three tablets of 50 mg per day. According to the expert opinions, omaveloxolone should be promptly discontinued if aspartate aminotransferase (AST) or alanine aminotransferase (ALT) increased to > 5 × upper limit of normal (ULN), or AST or ALT increased to > 3 × ULN, and bilirubin increased to > 2 × ULN. If/when levels would be stabilized or resolved, omaveloxolone might be reinitiated with an appropriate frequency of monitoring of liver function tests [10]. Informed consent was obtained from each participant.

### Clinical assessment

Patients were assessed with an anamnestic profile, and blood tests, at baseline (T0) and after 12 (W12) and 24 weeks (W24) of treatment. For each timepoint neurological examination and clinical scales were performed by two specialists in neuromuscular disorders (VDS and FB). Gait, cranial nerves, ocular movements, cerebellar function, superficial and deep sensitivity, proximal and distal muscle strength and osteotendinous reflexes in the four limbs were evaluated. Moreover, during clinical examinations, we assessed the modified FARS (mFARS), the Friedreich’s Ataxia Activities of Daily Living (FA-ADL), the SARA. The mFARS is a modified version of the FARS scale, designed to quantitatively evaluate the neurological severity of FRDA patients, providing a progression-sensitive tool for both natural history studies and clinical trials [11]. SARA is a clinical scale used to assess ataxia severity, comprising eight items—gait, stance, sitting, speech disturbance, finger chase, nose–finger test, fast alternating hand movements, and heel–shin slide—with a total score ranging from 0 (no ataxia) to 40 (most severe ataxia); limb kinetic functions are evaluated separately on both sides [12]. The FA-ADL is a clinician-administered, structured interview consisting of nine items that assess functional impairment in activities of daily living, including speech, eating, dressing, hygiene, falls, walking, sitting, swallowing, and bladder control [13]. Each item is scored on five predefined severity levels, with optional 0.5-point increments when deemed appropriate, and the total score ranges from 0 (no impairment) to 36 (severe impairment) [13]. In addition, we employed the Nine-Hole Peg Test (9HPT) to evaluate the upper limb dexterity.

### Laboratory analyses

We performed blood tests assessing blood count, renal function, thyroid function, AST, ALT, total, direct and indirect bilirubin, Gamma-glutamyl transferase (GGT), Alkaline phosphatase (ALP), total amylase, lipase, electrolytes, proteinogram, folates, B12 vitamin, N-terminal pro-B-type natriuretic peptide (NT-proBNP), creatine kinase (CK), lactate dehydrogenase (LDH), D-dimer, C reactive protein (CRP), serum iron, serum ferritin and serum transferrin, serum glucose, Interleukin-6 (IL-6), alpha-fetoprotein (AFP), Low-Density Lipoprotein cholesterol (LDL), High-Density Lipoprotein cholesterol (HDL), triglycerides, total cholesterol, fibrinogen, Prothrombin Time (PT), International Normalized Ratio (INR).

### Statistical analysis

Statistical analysis was performed by two qualified researchers (VDS and FB). Quantitative variables are reported in raw numbers and by calculating the mean, the standard deviation, and the percentages. Clinical scales and questionnaires were administered and compared at baseline and at follow-up at W12 and W24 through repeated measures of ANOVA. Similarly, laboratory measures were evaluated at baseline, W12 and W24. Responder rate was calculated considering a meaningful improvement of 2.5 points at the mFARS scale. Differences in clinical scales (SARA, mFARS and FA-ADL) were calculated between scores at W24 and T0 (ΔSARA, ΔmFARS and ΔFA-ADL). Correlation analysis was performed among clinical and laboratory data. Statistical significance was set at *p* < 0.05. Statistical analysis was performed with SPSS v26.

## Results

### Patients’ enrollment, demographic, and clinical characteristics at baseline

Twenty-nine patients with FRDA diagnosis were evaluated for study inclusion, but 9 of them were excluded as they were not eligible for treatment with omaveloxolone, because they presented an mFARS score ≥ 80 at study initiation. Hence, 20 patients (13 females, 65%) affected by FRDA with mean age of 40.6 ± 12.6 years and a duration of disease of 24.9 ± 9.5 years were treated with omaveloxolone and included in this study. Patients were followed-up for 25.2 ± 8.0 weeks after study inclusion. As expected, patients excluded from the study presented higher mFARS scores (*p* = 0.001), when compared to the ones enrolled, but there were no differences between the two groups depending on gender (*p* = 0.63), age (*p* = 0.34), age at onset (*p* = 0.18), and duration of the disease (*p* = 0.23). Table S1 (supplementary material) describes the clinical characteristics of the cohort.

### Data on efficacy

**Fig. 1:**
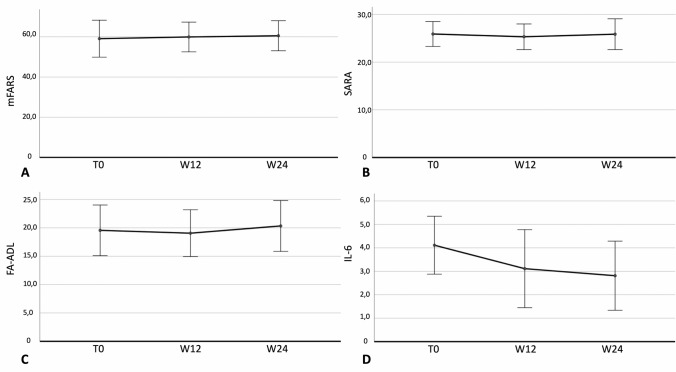
Clinical and laboratory data at baseline, W12, and W24 in 20 patients affected by Friedreich’s ataxia: mFARS (**A**), SARA (**B**), FA-ADL (**C**) and IL-6 levels (**D**). Abbreviations: W12, Week 12; W24, Week 24; FA-ADL, Friedreich Ataxia Activities of Daily Living; mFARS, modified Friedreich’s Ataxia Rating Scale; SARA, Scale for the Assessment and Rating of Ataxia; IL-6, Interleukin-6

During follow-up a subjective improvement was reported by patients without any signs or symptoms of progression. Figure 1 describes clinical data at baseline, W12, and W24.

No significant differences were reported during follow-up in the study cohort according to mFARS (*F* = 2.5; *P* = 0.15, Fig. 1A), SARA (*F* = 0.003; *P* = 0.96, Fig. 1B), FA-ADL (*F* = 0.33; *P* = 0.58, Fig. 1C), 9HPT at the right (*F* = 0.04; *P* = 0.84) and left (*F* = 1.4; *P* = 0.27). Conversely, IL-6 significantly decreased from T0 to W12 and W24 (*F* = 40.8; *P* < 0.0001, Fig. 1D). Table 1 describes the effect of omaveloxolone on clinical and laboratory data.

**Table 1:** Clinical and laboratory data at different timepoints

	Baseline	W12	W24	*P* value
FA-ADL	19.6 (5.8)	19.1 (5.4)	20.3 (5.8)	0.58
SARA	25.8 (4.3)	25.3 (4.4)	25.8 (5.3)	0.96
mFARS	59.1 (12.8)	59.9 (10.3)	60.5 (10.4)	0.15
9HPTr (seconds)	88.4 (33.1)	85.8 (32.0)	89.3 (35.9)	0.84
9HPTl (seconds)	126.4 (90.6)	106.3 (40.9)	100.0 (34)	0.27
CPK (U/L)	77.8 (34.7)	62.4 (25.9)	57.1 (38.2)	0.06
NT-proBNP (ng/L)	73.7 (85.1)	103.8 (121.7)	84.4 (96.3)	0.51
AST (U/L)	23.8 (9.8)	38.3 (14.9)	34.3 (26.8)	0.23
ALT (U/L)	32.0 (29.3)	77.1 (33.9)	47.5 (20.4)	0.15
TG (mg/dL)	126.5 (68.4)	126.8(74.1)	132.8 (59.4)	0.60
LDL (mg/dL)	129.3 (20.1)	138.8 (33.3)	129.3 (31.4)	1.00
CRP (mg/L)	2.3 (2.6)	2.8 (2.8)	1.8 (1.9)	0.08
IL-6 (pc/mL)	4.1 (1.6)	3.1 (2.2)	2.8 (1.9)	** < 0.0001**

*W12* Week 12, *W24* Week 24, *FA-ADL* Friedreich Ataxia Activities of Daily Living, *mFARS* modified Friedreich’s Ataxia Rating Scale, *SARA* Scale for the Assessment and Rating of Ataxia, *9HPr* 9-Hole Peg Test right, *9HPl* 9-Hole Peg Test left, *CPK* Creatine phosphokinase, *NT-proBNP* N-terminal pro-brain natriuretic peptide, *AST* Aspartate aminotransferase, *ALT* Alanine aminotransferase, *TG* triglycerides, *LDL* Low-Density Lipoprotein, *CRP* C-Reactive Protein, *IL-6* Interleukin-6

Each variable is expressed with mean and standard deviation with statistical significance with *p* < 0.05

### Responder analysis

Univariate and multivariate analyses were performed to identify possible factors associated with a response to omaveloxolone, defining a response as a reduction of 2.5 points to mFARS according to the MOXIE trial (Tables S2 and S3, supplementary material). After univariate analysis, CPK and NT-proBNP values seemed to be differently distributed among responders and non-responders to omaveloxolone (Table S2, supplementary material), but the effect disappeared at multivariate analysis (Table S3, supplementary material). Hence, no factors were identified as predictive of response to omaveloxolone were found.

### Safety and adverse events

Omaveloxolone was well-tolerated in all patients, and we did not experience any discontinuation after 24 weeks of follow-up. Considering the whole study sample, there were no significant modifications on laboratory exam during the follow-up with the only exception of IL-6. Moreover, 40% of patients did not report any adverse event. In 9 patients (45%) ALT elevation was reported, while only in one case AST increased. Of note, liver enzyme elevation was always transient and asymptomatic. Transient enzyme elevation was usually until × 2 the upper normal limit except for one young patient who presented an asymptomatic liver enzyme elevation × 10 after 12 weeks of treatment; in that case, after temporary drug discontinuation for 3-week, AST and ALT normalized, hence, the drug was started again after 3 weeks, without any significant further enzyme elevations nor signs of liver damage and the patients is still on treatment with omaveloxolone with 8 months of follow-up. No patients reported a clinically meaningful increase of cholesterol, triglycerides, nor cardiac biomarkers. Table 2 reports the adverse events encountered at the last follow-up in patients treated with omaveloxolone.

**Table 2:** Adverse events reported in 20 patients treated with omaveloxolone at the last available follow-up

Clinical variable	Total count (*N* = 20)
No adverse events	8 (40%)
Transient liver enzyme elevation	10 (50%)
Heartburn	3 (15%)
Diarrhoea	1 (5%)
Dysmenorrhoea	1 (5%)
Nausea	1 (5%)
Upper limb dysesthesia	1 (5%)
Urinary incontinence	1 (5%)
Urinary urgency	1 (5%)
Visual disturbances	1 (5%)

### Correlation analysis

9HPT on the right side correlated with SARA (*k* = + 0,52; *p* = 0.048), and mFARS (*k* = + 0,59; *p* = 0.017) and 9HPT on the left (*k* = + 0.88; *p* < 0.0001). 9HPT on the left side correlated with FA-ADL (*k* = + 0,72; *p* = 0.012), SARA (*k* = + 0,62; *p* = 0.014), and mFARS (*k* = + 0,71; *p* = 0.002). A positive correlation was found between FA-ADL and 9HPT on the left side (*k* = + 0,72; *p* = 0.012), but not with 9HPT on the right side (*p* = 0.083). In addition, a correlation was found between SARA and 9HPT both on the left (*k* = + 0,62; *p* = 0.014) and right side (*k* = + 0,52; *p* = 0.048).

Furthermore, considering the differences on clinical scales at W24 with respect to baseline, ΔmFARS was significantly positively correlated with time of exposure to omaveloxolone (*k* = + 0,92; *p* < 0.0001) and with FA-ADL at baseline (*k* = + 0,64; *p* = 0.048). In addition, ΔFA-ADL was correlated with IL-6 at baseline (*k* = + 0,67; *p* = 0.017). IL-6 was correlated with CRP (*k* = + 0,61; *p* = 0.004). Of interest cholesterol and triglycerides were not correlated with any variables.

## Discussion

This study represents an early real-life experience of adult patients affected by FRDA treated with omaveloxolone. FRDA, in the absence of therapy, is a neurodegenerative disease with an average progression in mFARS scores of approximately 2–2.2 points/year in adults, with more rapid progression in younger people and in the still ambulatory phase [14, 15]. In pediatric patients the scenario is even worse [16]. Although other drugs have been used off-label to counteract the currently known neurophysiological mechanism of FRDA, clinical data are still lacking. To date, omaveloxolone is the only specific drug for FRDA that, thanks to the multicenter, double-blind, placebo-controlled MOXIe study, has been shown to improve the course of the disease based on the mFARS scale. Specifically, in MOXIe part 1 with a 12-week follow-up, patients taking omaveloxolone recorded an improvement in the mFARS compared to baseline of − 3.8 points compared to baseline and of 2.3 compared to the control group [17]; in MOXIe part 2 with a 48-week follow-up, patients taking omaveloxolone recorded an improvement in the mFARS compared to baseline of − 1.55 ± 0.69 points, with a difference of − 2.40 ± 0.96 compared to the control group, which in turn recorded a worsening of + 0.85 ± 0.64 in the absence of drug. In the extended analysis of the same trial extended up to 144 weeks, a difference of − 2.91 ± 1.44 points was recorded compared to the control group and a mean change of 0 points compared to the 48-week follow-up, demonstrating maintenance of the initial clinical recovery over time [8]. However, real-world data on omaveloxolone in FRDA are still lacking, apart from single case reports [9].

In our prospective study, we evaluated the drug's effect on 20 FRDA patients followed up for 24 weeks of treatment in a real-life setting. These early data are in line with MOXIe trials might support the efficacy of omaveloxolone on stabilizing mFARS, SARA and FA-ADL scores, and the slowing effect of omaveloxolone on disease progression in FRDA. Indeed, no patient reported significant progression during treatment with no discontinuations at the last follow-up available. Moreover, the drug was safe with no significant adverse events during the first 24 weeks and without discontinuations. Indeed, although asymptomatic and transient liver transaminase elevation occurred in 50% of patients, but it did not cause any discontinuations nor symptoms or signs of liver damage confirming previously available data [10]. Of note, in all patients the entity of liver enzyme elevation was reduced after W12 and normalized at W24. Another point of discussion in FRDA patients treated with omaveloxolone is the potential cardiac toxicity as well as a protective long-term effect on cardiac progression or FRDA [18–20]. Cardiac function was stable, as confirmed also by stable NT-proBNP levels and lipidic metabolism was not altered by omaveloxolone. On this perspective, no significant worsening of symptoms with no signs of progression was reported in the absence of safety issues, confirming what expected by clinical trials in younger populations [7, 8, 19, 20]. Moreover, even in absence of significant clinical improvement on clinical scales, most patients reported a clinical subjective benefit, especially on the tone of voice and fatigue. Moreover, the fact that ΔmFARS was significantly positively correlated with the time of exposure to omaveloxolone and that ΔFA-ADL was correlated with IL-6 at baseline, which were reduced during treatment might suggest that a longer follow-up could highlight a measurable clinical improvement, but a longer observation is needed.

By the way, it is worth discussing that this cohort differs to the one of MOXIe trials; Indeed, the mean age of patients in this study sample is 41.27 ± 12.6 y with mean baseline mFARS of 60.42 ± 13.91, while the mean age in the part 1 MOXIe trial is 25.6 ± 6.5 y with a mean mFARS of 41.1 ± 11.5. On this perspective, our data extend the limited knowledge on omaveloxolone in adult FRDA patients with longer disease duration, poorly explored in clinical trials. This topic is particularly interesting considering the likely wider use of omaveloxolone in older patients in the next future [19]. Indeed, the drug is reimbursed without limits of age in patients with mFARS < 80 in Europe. Older patients with FRDA usually present an advanced state and high burden of disease [21], and this is the reason why we expect a positive but not striking effect of omaveloxolone. Indeed, selective structural damage to the dentate nucleus, as well as other cerebral and cerebellar areas may increase as the disease progresses thus limiting the functional reversibility. Omaveloxolone, as an Nrf2 activator, can improve the functioning of residual neurons, but the clinical effect might critically be connected to the neuronal reserve [22]. In our experience, we are observing a stabilization in older FRDA patients treated with omaveloxolone, but further studies are needed in FRDA subpopulations to confirm our preliminary data.

Finally, an interesting point of novelty of this study is a significant reduction in IL-6 levels starting at 12 weeks and lowering at 24 weeks. These data suggest an inflammatory involvement of cytokines and IL-6, as already reported in FRDA [23] supporting a possible immunomodulatory effect of omaveloxolone in adult FRDA patients. Furthermore, this interesting effect is hint by the significant positive correlation identified between ΔFA-ADL scores at W24 and IL-6 at baseline. However, this point needs further investigation and confirmation by future research.

## Strength and limitations

This observational study is, to our knowledge, the first cohort reporting real-life experience with omaveloxolone in FRDA. A strength of this study is the wide assessment with several clinical scales, as well as a deep evaluation of laboratory exams at single timepoint with not only liver function tests, but also cytokines, inflammatory and cardiac biomarkers. On this basis, the safety assessment could be considered very reliable, especially for liver function tests. However, this study carries several limitations: the relatively limited sample size, even considering the rarity of the disease, and the limited follow-up. On this perspective, the data reported should be considered preliminary. Indeed, it is difficult to enhance stabilization or improvement in FRDA patients based on such short follow-up in a small cohort. Further studies are needed to investigate the efficacy of omaveloxolone in FRDA.

## Conclusions

Omaveloxolone seems to be safe and well-tolerated in adult FRDA patients in the real-life setting. No neurological progression was reported with reduction of inflammatory biomarkers after 24 weeks of treatment, but no predictive factors for the disease response have been identified. However, the short duration, and the small sample size limit the generalizability of the results. Further multicentre studies with prolonged follow-up are needed to gather clinical experiences with omaveloxolone in FRDA.

## Supplementary Information

Below is the link to the electronic supplementary material.

Supplementary file1 (DOCX 25 KB)

## Data Availability

Data are available upon raisonable request to the corrisponding author.
