# Supplementary material for: Early experience on omaveloxolone in adult patients with Friedreich’s ataxia: a real-world observational study
Source: J Neurol. 2025 Nov 1;272(11):742. doi: 10.1007/s00415-025-13487-1 (PMC12579712; doi:10.1007/s00415-025-13487-1)
Supplement: Supplementary file 1 — Supplementary file1 (DOCX 25 KB) [file 415_2025_13487_MOESM1_ESM.docx]

**Supplementary materials**

**Table S1**. Characteristics of the cohort of 20 FRDA patients. M, male; F, female, FRDA, Friedreich Ataxia.

| PATIENTS | Sex | Age | Age at onset (years) | Onset | Duration (years) | Comorbities | Diabetes | Cardiopathy | Pes cavus | Gait impairment | Scoliosis | Follow-up (weeks) |
| --- | --- | --- | --- | --- | --- | --- | --- | --- | --- | --- | --- | --- |
| 1 | M | 46 | 15 | Ataxic gait | 32 | None | No | No | No | Yes | No | 24 |
| 2 | F | 40 | 13 | Ataxic gait | 28 | None | No | No | No | Yes | No | 25 |
| 3 | F | 57 | 28 | Undefined | 30 | Neurogenic bladder with overactive detrusor; cognitive impairment | No | No | Yes | Yes | No | 33 |
| 4 | M | 27 | 7 | Ataxic gait | 20 | None | No | No | No | Yes | No | 25 |
| 5 | F | 35 | 6 | Undefined | 30 | Hypertrophic cardiomyopathy | No | Yes | No | Yes | Yes | 30 |
| 6 | F | 27 | 21 | Undefined | 18 | Hypertrophic cardiomyopathy, lumbar disc disease | No | Yes | No | Yes | No | 24 |
| 7 | F | 37 | 18 | Ataxic gait | 20 | None | No | No | No | Yes | No | 16 |
| 8 | F | 41 | 18 | Ataxic gait | 24 | Arterial hypertension, Hashimoto's thyroiditis | Yes | No | No | Yes | No | 27 |
| 9 | F | 42 | 10 |  | 33 | None | No | No | No | Yes | No | 24 |
| 10 | F | 41 | 13 | Ataxic gait and dysarthria | 29 | None | No | No | No | Yes | No | 26 |
| 11 | F | 33 | 28 | Scoliosis | 6 | None | No | No | No | No | Yes | 18 |
| 12 | F | 61 | 40 | Ataxic gait and dysarthria | 22 | None | No | No | No | Yes | No | 28 |
| 13 | F | 47 | 13 | Ataxic gait and postural instability | 34 | Left ventricular hypertrophy | No | Yes | No | Yes | No | 29 |
| 14 | M | 35 | 11 | Ataxic gait | 24 | None | No | No | No | Yes | No | 27 |
| 15 | M | 70 | 46 | Ataxic gait | 25 | Arterial hypertension, dyslipidemia | No | No | No | Yes | No | 28 |
| 16 | F | 21 | 18 | Ataxic gait | 4 | None | No | No | No | Yes | Yes | 24 |
| 17 | M | 35 | 20 | Ataxic gait | 15 | Diverticulosis of the rectosigmoid colon, Benign prostatic hypertrophy | No | No | No | Yes | Yes | 28 |
| 18 | M | 55 | 24 | Ataxic gait | 42 | Heart failure, Cognitive-behavioral disorder with depressive psychosis, | No | Yes | No | Yes | No | 28 |
| 19 | M | 40 | 21 | Ataxic gait | 30 | None | No | No | No | Yes | No | 25 |
| 20 | F | 29 | 20 | Ataxic gait and coordination | 10 | None | No | No | No | No | No | 15 |

**Table S2.** Univariate analysis. Abbreviations: FA-ADL, Friedreich Ataxia Activities of Daily Living; SARA, Scale for the Assessment and Rating of Ataxia; mFARS, modified Friedreich’s Ataxia Rating Scale; CPK, Creatine phosphokinase; NT-proBNP, N-terminal pro-brain natriuretic peptide; CRP, C-Reactive Protein; IL6-, Interleukin-6. Continuous variables are expressed with mean and standard deviaton, while categorical with number count and percentages. Statistical significance was set at p<0.05.

|  | mFARS responders | mFARS non-responders | p-Value |
| --- | --- | --- | --- |
| Sex (males) | 6 (30%) | 14 (70%) | 0.96 |
| Disease Onset | 23.4 (15.4) | 17.8 (8.9) | 0.34 |
| Disease duration | 26.4 (13.1) | 24.3 (8.8) | 0.71 |
| FA-ADL T0 | 17.0 (13.0) | 17.1 (6.1) | 0.99 |
| SARA T0 | 25.3 (13.1) | 25.6 (4.1) | 0.94 |
| mFARS T0 | 65.0 (23.5) | 59.3 (9.5) | 0.46 |
| CPK T0 | 47.0 (10.5) | 82.9 (38.7) | **0.062** |
| CRP T0 | 1.15 (0.99) | 2.38 (2.33) | 0.28 |
| NT-proBNP | 226.9 (282) | 76.6 (82.8) | **0.09** |
| IL-6 | 4.2 (1.3) | 4.1 (1.7) | 0.91 |

**Table S3.** Multivariate analysis. Abbreviations: CPK, Creatine phosphokinase; NT-proBNP, N-terminal pro-brain natriuretic peptide. Continuous variables are expressed with mean and standard deviaton. Statistical significance was set at p<0.05.

|  | mFARS responders | mFARS non-responders | p-Value |
| --- | --- | --- | --- |
| Duration | 26.4 (13.1) | 24.3 (8.8) | 0.60 |
| CPK T0 | 47.0 (10.5) | 82.9 (38.7) | 0.059 |
| NT-proBNP | 226.9 (282) | 76.6 (82.8) | 0.10 |
| Age | 46.8 (15.6) | 39.0 (11.7) | 0.35 |
